# A Serological Investigation of Porcine Reproductive and Respiratory Syndrome and Three Coronaviruses in the Campania Region, Southern Italy

**DOI:** 10.3390/v15020300

**Published:** 2023-01-20

**Authors:** Gianmarco Ferrara, Emanuele D’Anza, Antonella Rossi, Elvira Improda, Valentina Iovane, Ugo Pagnini, Giuseppe Iovane, Serena Montagnaro

**Affiliations:** 1 Department of Veterinary Medicine and Animal Productions, University of Naples “Federico II”, Via Delpino 1, 80137 Naples, Italy; 2 Department of Agricultural Sciences, University of Naples “Federico II”, Via Università 100, 80055 Portici, Italy

**Keywords:** porcine epidemic diarrhea virus, transmissible gastroenteritis virus, porcine respiratory coronavirus, porcine coronaviruses, porcine reproductive and respiratory syndrome

## Abstract

Porcine coronaviruses and reproductive and respiratory syndrome (PRRS) are responsible for severe outbreaks that cause huge economic losses worldwide. In Italy, three coronaviruses have been reported historically: porcine epidemic diarrhea virus (PEDV), transmissible gastroenteritis virus (TGEV) and porcine respiratory coronavirus (PRCV). Although repeated outbreaks have been described, especially in northern Italy, where intensive pig farming is common, there is a worrying lack of information on the spread of these pathogens in Europe. In this work, we determined the seroprevalence of three porcine coronaviruses and PRRSV in the Campania region, southern Italy. A total of 443 samples were tested for the presence of antibodies against porcine coronaviruses and PRRSV using four different commercial ELISAs. Our results indicated that PEDV is the most prevalent among porcine coronaviruses, followed by TGEV, and finally PRCV. PRRSV appeared to be the most prevalent virus (16.7%). For coronaviruses, seroprevalence was higher in pigs raised in intensive farming systems. In terms of distribution, TGEV is more widespread in the province of Avellino, while PEDV and PRRSV are more prevalent in the province of Naples, emphasizing the epidemic nature of both infections. Interestingly, TGEV-positive animals are more common among growers, while seropositivity for PEDV and PRRSV was higher in adults. Our research provides new insights into the spread of swine coronaviruses and PRRSV in southern Italy, as well as a warning about the need for viral surveillance.

## 1. Introduction

Porcine reproductive and respiratory syndrome virus (PRRSV) was first described in the United States in 1987 as an enveloped, single-stranded RNA virus that causes one of the most serious diseases in the global swine industry [1]. PRRSV has a predilection for the respiratory and reproductive tracts and its symptoms include reproductive failure (abortion, return to estrus, stillbirth, premature farrowing) and respiratory symptoms. With the exception of some Scandinavian countries (Norway, Finland and Sweden) and Switzerland, the disease has been endemic in Europe since the 1990s [2]. Economic models estimate a median annual loss of EUR 127 to EUR 650 per sow in herds affected by this virus [3].

Coronaviruses (CoV) are the most widespread positive-sense and single-stranded RNA viruses that infect wild and domestic animals as well as humans. The scientific community’s increasing interest in these viruses is due to their relevant economic impact and potential interspecies transmission [4,5]. Coronaviruses generally have a strong propensity to recombine and mutate, often allowing them to cross and overcome the host species barrier. Recent examples of CoV spillover have been described, including severe acute respiratory syndrome coronavirus (SARS-CoV), middle eastern respiratory syndrome coronavirus (MERS-CoV) and the recent SARS-CoV 2 pandemic. Among animal coronaviruses, porcine coronaviruses are becoming increasingly important due to continuous epidemics occurring all over the world. Currently, six CoVs are known to be capable of infecting pigs [6]. Four of them belong to the alphacoronavirus genus—transmissible gastroenteritis coronavirus (TEGV), porcine respiratory coronavirus (PRCV), porcine epidemic diarrhea virus (PEDV), swine acute diarrhea syndrome coronavirus (SADS-CoV) —one to the betacoronavirus genus—porcine hemagglutinating encephalomyelitis virus (PHEV)—and one from to the deltacoronavirus genus—PDCoV [6]. Three of these (PEDV, TGEV and PRCV) have been described in European pigs [5]. PEDV is an RI-emerged alphacoronavirus responsible for acute enteric syndrome leading to malabsorption due to the atrophy of intestinal villus, diarrhea and vomiting [7]. PEDV is currently spreading throughout the world. Several outbreaks have been described over the years, including the one that occurred in the United States and China in 2010, which is known for its devastating economic impact [8,9,10,11]. Another extremely contagious alphacoronavirus, TGEV, was first isolated in the United States in 1946 before becoming widespread. PEDV and TGEV share many common features, including tropism, pathogenesis and clinical symptoms. TGEV outbreaks occur only sporadically nowadays because cross-protection is mediated by the less pathogenic PRCV [12]. Another CoV that infects the pigs is PRCV, a TGEV mutation identified in 1984 as the result of a deletion in the N-terminal portion of the spike protein. The virus was able to colonize pulmonary epithelial cells after this loss, which caused it to lose its affinity for intestinal cells. It currently primarily results in subclinical respiratory infections, the severity of which is largely determined by individual factors such as coinfections and immunodepression. PRCV was considered nonpathogenic, and most studies concluded that it only causes sub-clinical respiratory infections [5]. All three viruses are responsible for massive economic losses, as well as representing potential sources of future CoV recombination events.

In Italy, there is little information about these infections and evidence of viral circulation has been described mainly in the north of the country [13,14,15]. Knowledge of PRRS and CoVs epidemiology, as well as farm categorization based on disease status, is essential for developing effective control strategies for these diseases. Since information on the epidemiology of these viruses is completely lacking in the Campania region, the aim of this study was to fill this knowledge gap by demonstrating viral circulation using a serological approach, as well as identifying risk factors associated with a higher risk of infection.

## 2. Materials and Methods

### 2.1. Sampling

This survey was performed in the Campania region (410000000 N-143000000 E), southern Italy. Pig farming is not widely practiced in this region; in fact, at the time of sampling, approximately 75,000 pigs were bred, accounting for 2% of the Italian livestock (Banca Dati Nazionale dell’ Anagrafe Zootecnica, https://www.vetinfo.it/j6statistiche/, accessed on 15 November 2022). Because of the lack of further surveys in the same area, we opted for an expected prevalence of 0.5 (i.e., 50%), an absolute precision of 5% and a 95% confidence interval. Thrusfield’s formula was used to calculate the sample size in Epi Info:

*n* = Z^2^ × P(1 − P)/d^2^

where: Z = 1.96 for a confidence level of 95%, P = expected prevalence, d = 0.05 accepted error and *n* = sample size. A total of 438 blood samples from unvaccinated farms (32) were randomly collected concomitantly with blood collection by state veterinarians for the national pseudorabies eradication program (ethical approval was not required).

### 2.2. Enzyme-Linked Immunosorbent Assay

The presence of antibodies against porcine CoVs was determined using three commercial ELISAs: INgezim^®^ TGEV, INgezim^®^ PEDV and INgezim^®^ Differential coronavirus (Eurofins Ingenasa, Madrid, Spain. These tests are widely used in epidemiological studies to detect specific antibodies against PRRSV, PEDV and TGEV and to exclude any possible cross-reactions described between TGEV and PRCV, respectively. The presence of antibodies against PRRSV was determined using a commercial kit, (INgezim^®^ PRRS Universal, Eurofins Ingenasa, Madrid, Spain). All kits were used exactly as the manufacturers instructed. Optical density was measured with a spectrometer (Thermo Fisher Scientific, Life Technologies, Carlsbad, CA, USA) and used to calculate the cut-off value that distinguishes a positive from a negative sample.

### 2.3. Statistical Analysis

To assess the prevalence at the animal level, the number of positive pigs was divided by the total number of pigs investigated. Information obtained during the sample collection (location, age, sex and farm system) was used to analyze risk factors. Univariate analysis was performed at the animal level using chi-square statistics to identify risk factors for PEDV and TGEV positivity expressed as binary variables (PRCV was excluded due to the very low positive rate). A *p*-value lower than 0.05 was considered significant (MedCalc Statistical Software version 16.4.3, Ostend, Belgium). A map representing the spatial distribution of PEDV- and PRRSV-positive farms was designed using Epi Info.

## 3. Results

A total of 438 samples from 32 farms were tested for the presence of specific antibodies against three different porcine coronaviruses and PRRSV. Approximately 57.3% of the pigs tested were male (251/438), while 42.7% were female (187/438). The distribution among different provinces was as follows: 32.2% Avellino (141/438), 21.7% Benevento (95/438), 17.3% Caserta (76/438), 15.3% Napoli (67/438), 13.5% Salerno (59/438). A total of 324 animals were bred in intensive systems (74%) and the remaining 26% in extensive systems (114/438). Based on age and weight, 23.3% of the samples were collected from growers (10–18 weeks old and 8–25 kg of weight), 48% from finishers (18–26 weeks old and 50–105 kg of weight) and 28.7% from mature animals (>26 weeks old and >105 kg of weight). Descriptive information related to collected data was summarized in Supplementary Table S1.

**Figure 1:**
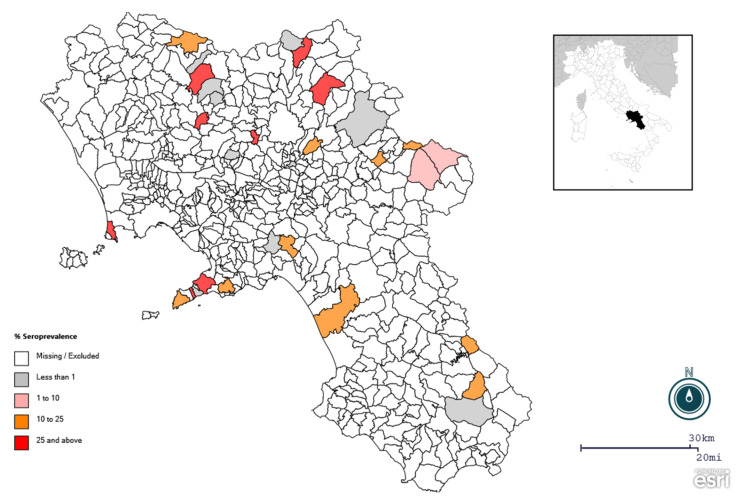
Spatial distribution of positive farms for PEDV in the pig population in the Campania region, southern Italy. A large proportion of the sampled districts had seroprevalence between 10% and 25% or higher.

**Figure 2:**
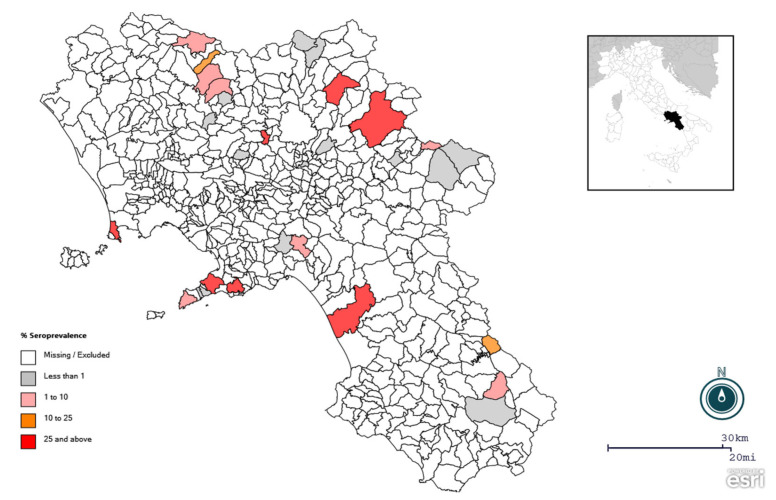
Spatial distribution of positive farms for PRRSV in the pig population in the Campania region, southern Italy. PEDV and PRRSV were found in high concentrations in several districts.

A total of 92 animals were seropositive for at least one porcine coronavirus (one sample was coinfected with TGEV and PEDV), with an apparent overall seroprevalence of 21%. Specific coronavirus seroprevalence was 14.8% for PEDV, 5.5% for TGEV and 0.9% for PRCV (Table 1). The highest seroprevalence was observed for PRRSV (16.7%). PEDV infection was revealed as the most prevalent CoV among the pig population in the Campania region. At the farm level, 22 of 32 were found positive for at least one porcine CoV, while the specific coronavirus herd seroprevalence was 68.7% for PEDV and 6.2% for TGEV and PRCV. Half of the farms analyzed tested positive for PRRSV (16/32). Since a very small number of positive animals were found for PRCV (*n* = 4), this virus was excluded from further statistical analysis. The univariate analysis (including four variables: sex, province, age, farming system) revealed non-significant differences between sex, while seroprevalence of PRRSV, PEDV and TGEV antibodies varied significantly among provinces (Table 2). In fact, higher seroprevalences of PRRSV and PEDV were observed in the province of Naples (56.7% and 29.8%, respectively), while a higher TGEV seroprevalence was obtained in the province of Avellino (17%) (Figure 1 and Figure 2). Higher seroprevalences of both CoVs were found in intensive farms (18.2% and 7.2% for PEDV and TGEV, respectively). TGEV seropositivity showed an age-related decreasing trend, as it was statistically higher in growers and tended to decrease in finishers and adults (Table 2). The reverse trend, although not statistically significant, was observed for PEDV. Higher PRRSV seroprevalence was obtained in adult animals (26.2%) (Table 3).

## 4. Discussion

Despite frequent outbreaks in Europe, reports of the occurrence of porcine coronaviruses in Italy are confined to northern Italy, whereas there is no information on the distribution of these viruses in the domestic pig population in southern Italy. The same could be stated for PRRSV, whose seroprevalence in pig populations was reported to be 24.7% in northern Italy and 37% in the wild boar population in southern Italy [16,17]. The detection of antibodies against porcine CoVs was carried out only during the PEDV outbreaks that occurred in northern Italy, revealing 22/40 seropositive animals among symptomatic pigs during the first outbreak (2005–2006) and a 92% seropositive rate during the second wave (2007–2014) [13,18,19]. Our study represents the first serosurvey of three CoVs in Italy and draws attention to the spread of CoVs among domestic pigs, reporting an overall seroprevalence of 22%. The specific seroprevalences found were consistent with those seen in other European countries and with historical periods. Indeed, there has been a reported decline of TGEV (due to PRCV infection, which is less pathogenic and has short-lived naturally detectable antibodies) and a rise in PEDV infection in recent years [12,20]. The highest seroprevalence so far reported worldwide in an apparent health population has been described in Asia. A recent work described 96.7% of seropositive animals among the domestic pig population in northern Vietnam [21]. Also, in Asia, there was a seroprevalence of 39.56% among Tibetan pigs in China in 2015 [22]. In Europe, lower seroprevalences have been described. For example, in the Netherlands, during a comprehensive study of 838 domestic pigs, only nine tested positive in ELISA (1.07%), and only 2 (0.24%) were confirmed in virus neutralization [23]. A post-outbreak serological survey made in eastern Croatia, revealed antibodies against PEDV in 15.62% of tested pigs, most of them bred on large industrial farms [24]. In Poland, antibodies against PEDV were detected in 3.2% of tested wild boars [25]. As for PRRSV, high seroprevalences are also described in other European countries, reaching levels between 20 and 60% [2,26,27].

Global data on TGEV and PRCV Ab-prevalence is limited, and the results are mostly shown as negative or show low seroprevalence rates in both domestic pigs and wild boars, as described in Argentina, Slovenia, Finland, the Czech Republic, Germany, Croatia and Turkey [28,29,30,31,32,33,34]. Exceptions have been described in South Korea, where a PRCV seroprevalence of 53.1% was described in 1999, and in Hungary, where a seroprevalence of 15.42% against TGEV was reported in 2019, even though most of the animals proved to be positive for PRCV [35,36]. An extensive study was performed in Japan in 2010, obtaining a seroprevalence of 14.4% after testing 2703 pig sera for TGEV antibodies [37]. A recent study conducted in the same region (Campania) on wild boar populations showed that PEDV was the most prevalent CoV in wild boar, with a seroprevalence of 3.83%, while a negligible seroprevalence of 0.67% was found for TGEV and PRCV [38]. The findings obtained in the present study confirmed the same in domestic pigs.

Univariate analysis revealed that the seroprevalence of PEDV and TGEV was higher in pigs kept in intensive farming systems. We may even assume that the spread of these viruses is wider in domestic pigs than in wild boars due to density; in fact, farm size and animal density have been demonstrated to have a role in disease transmission. There were no gender differences detected, which is also supported by further research, distinguishing this virus from others, in which female subjects are more exposed according to ethological reasons [21,22,39].

As observed in other studies, some sub-areas (Avellino province for TGEV and Naples province for PEDV) showed higher seroprevalences, possibly due to the epidemic nature of porcine CoVs [21,22,24]. Lower seroprevalences were found in the province of Caserta (18.2% and 0% for PEDV and TGEV, respectively), where the Casertana breed, an autochthonous breed raised half-wild, is primarily kept in extensive systems with grazing opportunities [40]. Typically, this type of system promotes the spread of pathogens and the transmission of infections such as pseudorabies, tuberculosis, brucellosis, circovirus and others from wild boar populations to domestic pigs and vice versa [39,41,42,43]. Our data, coupled with those on the diffusion of CoVs in the wild boar population in the same study area, suggest that CoVs are more prevalent in pigs.

TGEV-positive animals are more common in growers, with an age-related decreasing trend. This trend was confirmed by Valkò et al., who did not find positive sows [35]. On the other hand, PEDV seropositivity was greater in adult animals (even if not statistically significant). Gao et al. and Myint et al. reported higher positive rates in young animals and a decline in PEDV seroprevalence among the age classes of juveniles, subadults and adults in previous studies, possibly due to the short period of detectable antibodies after primary infection [21,22]. This could be attributable to epidemiological differences among the strains, in addition to differences in sample, technique and methodology. The absence of neonatal infection during PEDV infection was a significant difference between PEDV and TEGV infection, even though this virus was later reported as pathogenic for neonatal and suckling pigs, dramatically increasing its economic impact [20,44]. We could hypothesize that this feature is not common among the different PEDV strains circulating across the world [44]. It has been reported that the number of sows played a role during the past European outbreaks [19,20]. In a longitudinal study conducted in Italy, 54% of the sows had anti-PEDV antibodies, but only 3% of piglets showed detectable antibodies (as well as a lack of clinical signs at 3–6 days of age during the last outbreak) [19]. Moreover, during the 2008–2014 outbreaks, the disease was observed mainly in grower and finisher herds [13].

Univariate analysis of risk factors involved in PRRSV seropositivity revealed an absence of statistical association for gender, while we found higher seroprevalences in adult animals. Highly positive farms clustered in the province of Naples. We found 21 of the total 22 coinfected PEDV-PRRSV animals in this province. Further investigations based on molecular approaches should be extremely useful in determining the prevalent strain or genotype of PEDV and PRRSV in southern Italy.

Our research provides new knowledge regarding the spread of swine coronaviruses and PRRSV in southern Italy, as well as a warning about the need for viral surveillance. The results we observe may be the result of a past epidemic or may even be an alert for future epidemics. PED is becoming endemic and regressing in Europe, as evidenced by the disappearance of antibodies reported in several countries after new outbreaks. It is difficult to understand why, in the absence of any special control measures, PEDV has been showing this trend in Europe [9,20]. The mechanisms behind the regression/waning of PEDV in Europe remain a big question mark. We could hypothesize that the lack of immunity and the increase in susceptible-seronegative pigs, as a result of the cyclic disappearance of antibodies, promote viral circulation to the point where outbreaks occur [6,7]. The epidemiology of PED in Europe has been and continues to be puzzling, and only accurate and continuous surveillance activity can help the authorities control these important infections.

## 5. Conclusions

Our study is the first to perform a serosurvey of three CoVs in Italy, and it highlights the prevalence of CoVs in domestic pigs, with an overall seroprevalence of 22%. According to the specific seroprevalences, PEDV is the most common CoV in the pig population in southern Italy. After several years, a study has revealed new information about the spread of PRRSV in Italy. Our findings enrich our understanding of the spread of coronaviruses and PRRSV in pigs in southern Italy, and remind us of the importance of viral surveillance.

## Figures and Tables

**Table 1:** Seroprevalence of porcine and reproductive syndrome virus and three different porcine CoVs in the pig population in the Campania region, southern Italy.

Factor	*n*	Positive	%	95%CI	χ^2^	*p*
Porcine coronavirus						
PEDV	438	65	14.8	11.5–18.2		
TGEV	438	24	5.5	3.3–7.6	67.14	<0.0001
PRCV	438	4	0.9	0–1.8		
Total	438	92	21	17.2–24.8		
Porcine reproductive and respiratory syndrome virus						
PRRSV	438	73	16.7	13.2–20.2	86.7	<0.0001

**Table 2:** Univariate analysis (chi-square) of a potential risk factor for PEDV and TGEV seropositivity.

	PEDV						TGEV					
Factor	*n*	Positive	%	95% CI	χ^2^	*p*	*n*	Positive	%	95% CI	χ^2^	*p*
Total	438	65	14.8	11.5–18.2			438	24	5.5	3.3–7.6		
Province												
Avellino	141	10	7	2.9–11.3			141	24	17	10.8–23.2	53.48	<0.001
Benevento	95	15	15.8	8.5–23.1			95	0	0	0		
Salerno	59	7	11.9	3.6–20.1			59	0	0	0		
Caserta	76	14	18.4	9.7–27.1			76	0	0	0		
Napoli	67	20	29.8	18.9–40.8	19.62	<0.001	67	0	0	0		
Sex												
Male	251	35	13.9	9.7–18.2			251	15	6	3–8.9		
					0.58	0.45					0.28	0.59
Female	187	31	16.6	11.2–21.9			187	9	4.8	1.7–7.9		
Age												
Growers	102	10	9.8	4–15.6			102	0	0	0		
Finishers	210	30	14.3	9.5–19	5.36	0.07	210	11	5.2	2.2–8.2	17.72	<0.001
Mature	126	26	20.6	13.6–27.7			126	13	12.7	5–15.6		
Farming system												
Intensive	324	59	18.2	14–22.4			324	24	7.4	4.5–10.3		
					9.6	0.002					8.9	0.003
Extensive	114	7	6.1	1.7–10.5			114	0	0	0		

**Table 3:** Univariate analysis (chi-square) of a potential risk factor for PRRSV seropositivity.

	PRRSV					
Factor	*n*	Positive	%	95% CI	χ^2^	*p*
Total	438	73	16.7	13.2–20.2		
Province						
Avellino	141	10	7.1	2.9–11.3		
Benevento	95	16	16.8	9.3–24.4		
Salerno	59	5	8.5	1.4–15.6		
Caserta	76	4	5.3	0.2–10.3		
Napoli	67	38	56.7	44.8–68.6	96.6	<0.001
Sex						
Male	251	46	18.3	13.5–23.1		
					1.16	0.28
Female	187	27	14.4	9.4–19.5		
Age						
Growers	102	3	2.9	0–6.2		
Finishers	210	37	17.6	12.5–27.8	22.2	<0.001
Mature	126	33	26.2	18.5–33.9		
Farming system						
Intensive	324	56	17.3	13.2–21.4		
					0.34	0.56
Extensive	114	17	14.9	8.4–21.4		

## Data Availability

Not applicable.

## Supplementary Materials

The following supporting information can be downloaded at: https://www.mdpi.com/article/10.3390/v15020300/s1. Table S1: Summary regarding the descriptive information of collected data.
