# Supplementary material for: A Serological Investigation of Porcine Reproductive and Respiratory Syndrome and Three Coronaviruses in the Campania Region, Southern Italy
Source: Viruses. 2023 Jan 20;15(2):300. doi: 10.3390/v15020300 (PMC9964103; doi:10.3390/v15020300)
Supplement: Supplementary file 1 [file viruses-15-00300-s001.zip › viruses-2158442-supplementary.pdf]

Supplementary table 1: Summary regarding the descriptive information of collected data.

Factor	n	%
Total	438	100
Province		
Avellino	141	32.2
Benevento	95	21.7
Salerno	59	13.5
Caserta	76	17.3
Napoli	67	15.3
Gender		
Male	251	57.3
Female	187	42.7
Age		
Growers	102	23.3
Finishers	210	48
Mature	126	28.7
Farming system		
Intensive	324	74
Extensive	114	26
